# FCIQMC-Tailored Distinguishable Cluster Approach: Open-Shell Systems

**DOI:** 10.1021/acs.jctc.2c00059

**Published:** 2022-05-06

**Authors:** Eugenio Vitale, Giovanni Li Manni, Ali Alavi, Daniel Kats

**Affiliations:** † Max Planck Institute for Solid State Research, Heisenbergstraße 1, 70569 Stuttgart, Germany; ‡ Department of Chemistry, University of Cambridge, Lensfield Road, Cambridge CB2 1EW, United Kingdom

## Abstract

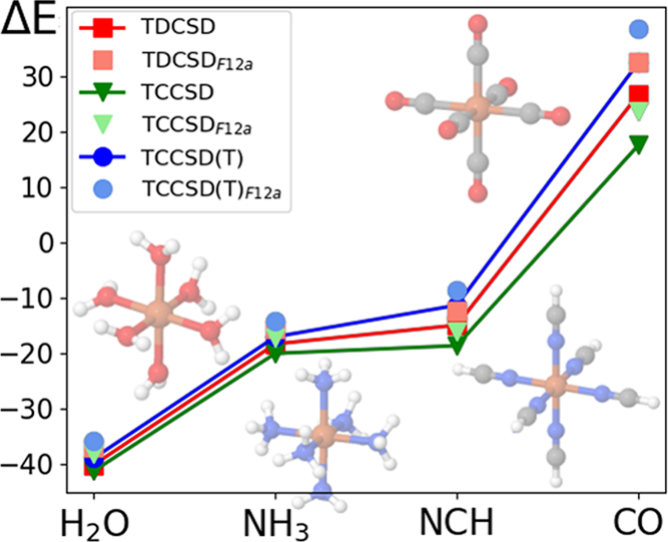

A recently proposed tailored approach based on the distinguishable cluster method and the stochastic FCI solver, FCIQMC [*J. Chem. Theory Comput*. 2020, 16, 5621], is extended to open-shell molecular systems. The method is employed to calculate spin gaps of various Fe(II) complexes, including a Fe(II) porphyrin model system. Both distinguishable cluster and fully relaxed CASSCF natural orbitals were used in this work as reference for the subsequent tailored distinguishable cluster calculations. The distinguishable cluster natural orbitals occupation numbers were also used as an aid to the selection of the active space. The effect of the active space sizes and of the explicit correlation correction (F12) onto the predicted spin gaps is investigated. The tailored distinguishable cluster with singles and doubles yields consistently more accurate results compared to the tailored coupled cluster with singles and doubles.

## 1 Introduction

Accurate and reliable computation of spin gaps of transition-metal complexes represents a challenge for quantum chemical methods.^1^ The correct estimation of the relative stability of spin states is important not only to identify the right ground state but also because reactivity patterns in catalytic and enzymatic processes are deeply influenced by the spin gaps. Density functional theory (DFT) is often employed to study these systems,^2−5^ but the results can drastically change depending on the choice of the functional,^4−9^ with differences up to 20 kcal/mol, which obviously is very problematic if the spin gap itself is only a few kcal/mol. Wave function theory approaches are, in general, computationally more expensive but can offer higher accuracy and systematic improvability. The coupled-cluster (CC) theory^10−12^ is popular for its hierarchy of methods, which rapidly converges to the full configuration interaction (FCI) limit for weakly correlated systems. The most common CC method includes only singles and doubles amplitudes (CCSD),^13^ but higher excitations must be taken into account to achieve chemical accuracy. The obvious further step includes triples, CCSDT, which quickly becomes prohibitively expensive and can be applied only to small systems. A more practical version with perturbative triples (CCSD(T))^14^ is usually adopted. Although these truncated CC methods are very accurate for a wide range of molecular systems, they are still based on a single-reference (SR) formalism, and typically fail for strongly correlated systems. The distinguishable cluster (DC) approach^15−19^ has been established in the last decade as a convenient way to improve the results for such systems without increasing the computational cost. However, the SR framework is not well suited to recover the complexity of wave functions with multiple similarly weighted most important configurations. These systems are usually studied by multireference (MR) approaches.

The complete active space self-consistent field (CASSCF) method^20−25^ is typically the first step of common MR strategies used in molecular systems. In this method, an active orbital space is selected and the electron correlation in this space is recovered at the FCI level. Simultaneously, the orbitals are relaxed under the mean field generated by the multiconfigurational many-body expansions. The exponential scaling of the FCI method limits the active space sizes for conventional CASSCF to ∼18 electrons in 18 orbitals. Substantial advances have been made in the past decades to mitigate the exponential scaling of FCI (and CASSCF), and various approximate methods have been developed, e.g., density matrix renormalization group (DMRG)^26−32^ or FCI Quantum Monte Carlo (FCIQMC),^33−37^ and the corresponding CASSCF methods, DMRG-SCF^38−40^ and Stochastic-CASSCF.^41^ Additional truncations of the active space can further reduce the computational cost, as is done in generalized active space (GAS),^42,43^ GASSCF,^44^ restrict-CASSCF in Molpro,^45^ ORMAS^46^ from GAMESS,^47^ and Stochastic-GASSCF^48,49^ approaches.

In the CASSCF approach, the electron correlation outside of the active space is missing, and therefore the results are usually only qualitatively correct. Higher accuracy can be achieved by employing methods that recover the missing part of the correlation on top of CASSCF or GASSCF, e.g., CAS second-order perturbation theory (CASPT2)^50,51^ or GASPT2,^52^ multireference configuration interaction (MRCI),^53^ multireference coupled cluster (MRCC),^54^ or multiconfiguration pair-density functional theory (MC-PDFT).^55−58^

The SR CC methods can also be used to recover dynamic correlation outside of the active space. In this respect, externally corrected CC methods^59−68^ utilize information from the active space to improve the CC description, without leaving the simple and comparably inexpensive SR framework. The tailored CC (TCC) approach introduced by Kinoshita et al.^69^ is one of these methods. In this approach, the singles and doubles amplitudes in CAS are frozen at the FCI level and all other amplitudes are optimized using CC amplitude equations. It has been combined with the CCSD and CCSD(T) methods, yielding tailored CCSD (TCCSD) and TCCSD(T).^70^ Recently, we have combined the tailored approach with distinguishable cluster with singles and doubles (DCSD), which has been demonstrated for closed-shell systems to yield substantially more accurate results than TCCSD.^71^ Two main drawbacks of tailored approaches exist, the missing relaxation of the CAS amplitudes and the fact that the tailoring is still inherently single reference. Both limitations are to some extent alleviated by enlarging the active space.^72,73^ We use FCIQMC to obtain the approximate FCI solutions in large active spaces. The active space can be specified either using (Stochastic-) CASSCF or through DCSD natural orbitals (NOs), as demonstrated in the previous publication.^71^ However, the tailored methods are useful not only to describe molecular systems with a large amount of static electron correlation. Also studies on systems with mostly dynamical electron correlation, but which require high-level single-reference methods for an accurate description, can benefit from the tailored methods. A typical example of such studies is the computation of spin gaps of single-center transition-metal complexes.^74−89^

In this study, the TDCSD method is extended to open-shell molecular systems and employed to calculate spin gaps of various iron(II) complexes. The accurate computation of spin-state splittings of such compounds is not a trivial task. These systems are not characterized by a strong MR character. However, dynamic correlation effects are important and different for each spin state, and neglecting them leads to large errors in the resulting spin-gap predictions. Thus, they represent an interesting subject to assess the validity of our novel FCIQMC-TDCSD approach.

## 2 Theoretical Overview

In the following, a brief overview of the computational methods employed in FCIQMC-tailored DCSD is given. Further theoretical details can be found in the previous publication.^71^

The tailored DC (TDC) and TCC methods have been implemented in the Molpro package,^45,90,91^ and the extraction of the CI coefficients from FCIQMC has been implemented in the NECI program.^92^

### 2.1 Distinguishable Cluster

The DC approach is a small modification of the amplitude equations in the CC with doubles (CCD). The intercluster exchange diagrams are removed, and the remaining quadratic terms are rescaled to restore the particle-hole symmetry of the amplitude equations and the exactness for two electrons (after orbital relaxation).^15,93^ A partial orbital relaxation can be achieved by introducing single excitations using the *e*^*T̂*_1_^ similarity-transformed Hamiltonian.^16^ The resulting DCSD method is size-extensive, orbital-invariant with respect to intraspace orbital rotations, and as insensitive to the interspace orbital rotations as CCSD.

DCSD has been shown in numerous benchmarks to be more accurate than CCSD for weakly correlated systems.^17^ Furthermore, it vastly outperforms CCSD for strongly correlated systems, often yielding qualitatively good results in situations where the traditional CC breaks down.

One-body density matrices can be calculated in the usual manner using the Lagrange technique, and natural orbitals and occupation numbers are obtained as eigenvectors and eigenvalues of such matrices, respectively.

The basis set incompleteness error is largely reduced by adopting a perturbative basis set correction (denoted by a subscript *F*12*a*)^94,95^

### 2.2 FCI Quantum Monte Carlo

The FCIQMC method is used to perform stochastic FCI calculations on very large active spaces and molecules consisting of several atoms. In this work, the corresponding Hilbert spaces have been spanned by Slater determinants, which are stochastically sampled and populated by signed walkers. The dynamics of the walker population evolves according to the imaginary-time Schrödinger equation
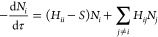
1where *N*_*i*_ is the number of walkers on the determinant *i*, τ is the imaginary time, *H*_*ij*_ are the Hamiltonian matrix elements in the basis of Slater determinants, and *S* is a shift parameter that controls the total walker number. As long as a sufficient number of walkers is provided, a long time integration of the imaginary-time Schrödinger equation converges to the ground-state wave function. The initiator method (i-FCIQMC)^34^ helps to dynamically truncate the Hilbert space (and the adaptive shift approach^96,97^ drastically reduces the associated error) extending the applicability of FCIQMC to even larger molecules.

Additionally, the stochastic noise can be reduced using the semistochastic approach,^98,99^ where a number of determinants are treated deterministically in the imaginary-time propagation.

FCIQMC has been combined with the Super-CI algorithm to obtain the Stochastic-CASSCF,^41^ which can be utilized to perform large-active-space CASSCF calculations.

Finally, the combination of FCIQMC with CC has already been studied in the last few years with different objectives to our FCIQMC-TDC approach (see refs (100−102)).

### 2.3 FCIQMC-TDC

The basic concept of tailored methods is based on the split-amplitude ansatz, which is straightforward for the CC wave function
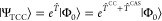
2The *T̂*^CAS^ cluster operators with amplitudes extracted from an external calculation represent the strongest part of the electron correlation in the system, and the rest of the cluster operators, *T̂*^CC^, is responsible for the remaining weaker dynamic correlation. The *T̂*^CAS^ amplitudes are obtained using well-known relations from CI coefficients, which are extracted and averaged from FCIQMC calculations inside of the active space. *T̂*^CAS^ amplitudes are kept frozen in the tailored calculation, and only the *T̂*^CC^ amplitudes are optimized. The error introduced by missing the relaxation of *T̂*^CAS^ can be reduced by enlarging the active space. As demonstrated in our previous publication,^71^ the tailored approach can also be applied to modified coupled-cluster methods, in particular DCSD, greatly enhancing the accuracy without an increase of the computational cost.

As in other embedding or active-space-based methods, a correct selection of the active space for the external correction is essential for accurate results. Here, we employ the (Stochastic-)CASSCF method to optimize the active space. For large active spaces, we also utilize DCSD NOs to define the active space, which has been shown to be comparably accurate to the CASSCF-defined CASs in our previous studies.^71^

The tailored methods still retain a strong dependence on a particular reference determinant. However, thanks to FCIQMC, calculations for large active spaces are possible, which drastically reduces the potential error caused by the bias toward a specific determinant.

Additionally, we have extended our FCIQMC-TCCSD implementation to include the perturbative triples correction, FCIQMC-TCCSD(T), first introduced by Lyakh et al.^70^ The perturbative triples (T) correction depends only on the *T̂*^CC^ amplitudes, and the computational cost is the same as of CCSD(T).

Finally, a perturbative basis set correction is also applied to our tailored approaches (TDCSD_*F*12*a*_, TCCSD_*F*12*a*_, and TCCSD(T)_*F*12*a*_), as also described in our previous publication.^71^

## 3 Results

### 3.1 Computational Details

The accuracy of the FCIQMC-TDC approach for open-shell systems has been evaluated by computing electronic spin states of five iron(II)-complexes. This test set includes four Fe(II) octahedral complexes, [Fe(H_2_O)_6_]^2+^, [Fe(NH_3_)_6_]^2+^, [Fe(NCH)_6_]^2+^, and [Fe(CO)_6_]^2+^, which have been studied previously with various methods,^74−86^ as well as a four-coordinated ferrous porphyrin model.^48,87,88,103−105^ The range of spin gaps goes from tens of kcal/mol for the former systems to a few kcal/mol for the iron-porphyrin model.

All tailored calculations included in this study have been done on top of NOs, either from (Stochastic-)CASSCF or from DCSD calculations, and all iron(II)-complexes have been evaluated at the CC level including the semicore orbitals of Fe (3s3p shell).^87,88^ The basis set used for the Fe(II)-porphyrin is the generally contracted atomic natural orbitals ANO-RCC-VTZP, while for the remaining four complexes, cc-pVTZ was employed.

All of the FCIQMC calculations performed in our study utilize the semistochastic approach with a deterministic space of up to 10^6^ Slater determinants. Additionally, the adaptive shift method^96^ has been used in all our calculations, and the trial space that defines our new shift *S* was composed of 1000 determinants. The number of walkers used in our FCIQMC was up to 2 × 10^8^, adjusted according to the sizes of the studied CASs.

For comparison, we have also computed DCSD, CCSD, and CCSD(T) estimates of the spin gaps using restricted open-shell Hartree–Fock (ROHF) orbitals, and CASPT2 and MC-PDFT on top of the CASSCF wave functions. The MC-PDFT values are obtained with the tPBE translated functional, which was shown to perform well on the prediction of spin-state ordering with a substantially lower cost compared to CASPT2.^81^ Although the DCSD and CCSD results are reported without F12 correction, we estimate that the latter will be similar to the correction obtained for the corresponding tailored approaches. All of the spin gap values calculated in this work are listed in the Supporting Information.

The usual convention to define the size of the active space, (*n*_electrons_, *n*_orbitals_), is employed.

### 3.2 Small Ligand Fe(II) Complexes

Energy spin gaps of four transition-metal complexes in an octahedral environment have been computed to test the accuracy of the FCIQMC-TDC and FCIQMC-TCC approaches for open-shell systems. These compounds, which can be written as [Fe(L)_6_]^2+^ (L = H_2_O, NH_3_, NCH, and CO), have been the focus of many different studies over the years.^74−86^ In the present work, we focus on the singlet (low spin, LS) and quintet (high spin, HS) spin gaps, given by Δ*E* = *E*_HS_ – *E*_LS_. The main computational challenge of these Fe(II) complexes lies in a balanced description of the dynamic correlation across spin states. An accurate sub-kcal/mol description often requires high-level methods beyond the “gold standard” CCSD(T).^105^

In this work, the spin gaps of the four octahedral Fe(II)-complexes have been investigated by varying the active spaces and using different methods. The small active spaces, CAS(6,5) and CAS(6,10), have been optimized at the deterministic CASSCF level. The former CAS comprises the five 3d orbitals of Fe together with six electrons, whereas in the latter CAS, the five correlating d′ orbitals are added to the CAS(6,5), to take into account the double-shell correlation of 3d orbitals. The larger active spaces considered for these four compounds include over 30 electrons in more than 30 orbitals, and have been defined by DCSD NOs corresponding to occupation numbers deviating strongly from 0 and 2. Additionally, we ensure that both spin states under consideration have a similar active space, i.e., if some type of orbitals is important for one of the spin states, it is included in both spin states. The orbitals chosen in the various CASs are available in the Supporting Information. Subsequently, FCIQMC calculations within the active spaces have been performed to obtain the CI vectors.

The geometries of these octahedral systems and diffusion Monte Carlo (DMC) results are taken from a work of Song et al.^82^

#### 3.2.1 [Fe(H_2_O)_6_]^2+^

Our first test system is the Fe(II) water–ligand complex. As discussed in Section 3.2, three different active spaces have been evaluated using various methodologies. The large active space contains a total of 42 electrons and 41 orbitals, CAS(42,41), as defined by the most correlated DCSD NOs, and comprises 3d orbitals of the iron atom, and various σ and π type orbitals (see the Supporting Information).

**Figure 1:**
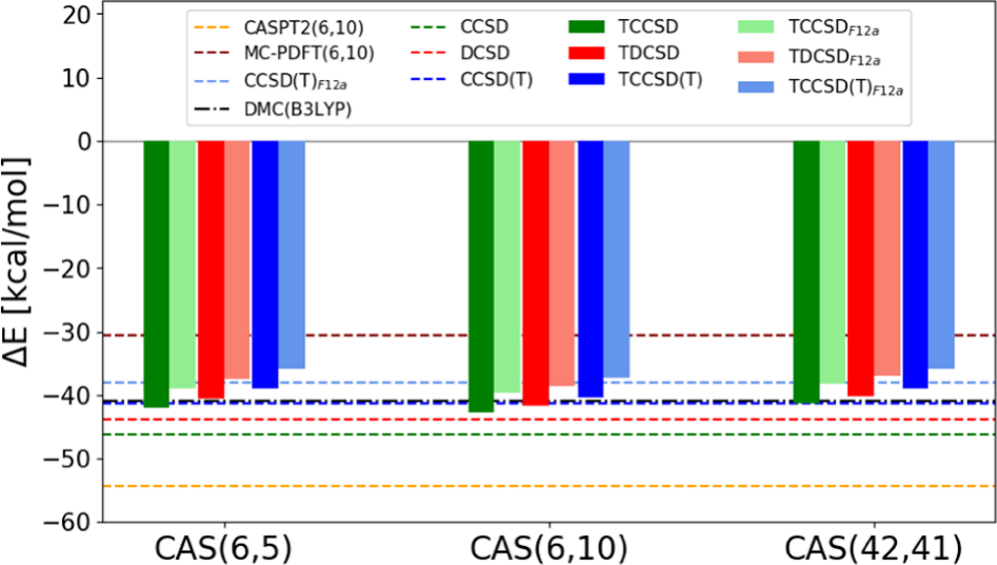
Spin gaps of [Fe(H_2_O)_6_]^2+^ over different active spaces.

For this compound, a high-spin ground state is expected. Thus, not surprisingly, already ROHF alone yields a correct order of states (Δ*E*_ROHF_ = −78.8 kcal/mol). However, ROHF overstabilizes the HS state. A reduction of the energy gap is predicted by correlated methods. The tailored methods yield consistent results across the different active spaces and in good agreement with ROHF-based coupled-cluster calculations, DMC and MC-PDFT (Figure 1).

The largest doubles amplitude in CAS(42,41), which corresponds to the second largest CI coefficient after intermediate normalization and which can be used as a measure of the multiconfigurationality of the system, is 0.041 for the LS state and 0.038 for the HS state. Thus, this compound is mostly single-configurational.

For all tailored cases, a perturbative F12 basis set correction has been evaluated. It is presented in Figure 1 with lighter colors relative to the underlying methods. The basis set correction reduces the spin gap further, bringing the results closer to the MC-PDFT(6,10) value and in general agreement also with CCSD(T)_*F*12*a*_. Interestingly, CASPT2 and the low-order CCSD and DCSD methods overstabilize the HS state, most certainly due to the lack of the higher-order many-body effects, as discussed elsewhere.^104^ On the contrary, the perturbatively corrected CCSD(T) method, DMC, TCC (especially upon F12 correction), and MC-PDFT are capable of capturing these forms of weak correlation and stabilize the LS state, reducing the spin gap. The TCCSD(T)_*F*12*a*_ result lies in between the MC-PDFT and the DMC predictions. For this system, the size and quality of the active space have only a negligible effect on the TCC predictions.

#### 3.2.2 [Fe(NH_3_)_6_]^2+^

Next, we evaluate the spin gap estimate of the [Fe(NH_3_)_6_]^2+^ complex. Again, the small CASs have been optimized at the CASSCF level and include 3d and d′ orbitals, and the large CAS obtained from DCSD NOs contains 42 electrons in 41 orbitals (see the Supporting Information).

As in the previous case, a high-spin ground state is observed, and electron correlation reduces the spin gap, which can be seen already at the CASSCF (or CAS-CI) level: Δ*E*_CASSCF(6,5)_ = −60.9 kcal/mol; Δ*E*_CASSCF(6,10)_ = −48.1 kcal/mol; Δ*E*_CAS-CI(42,41)_ = −40.0 kcal/mol.

**Figure 2:**
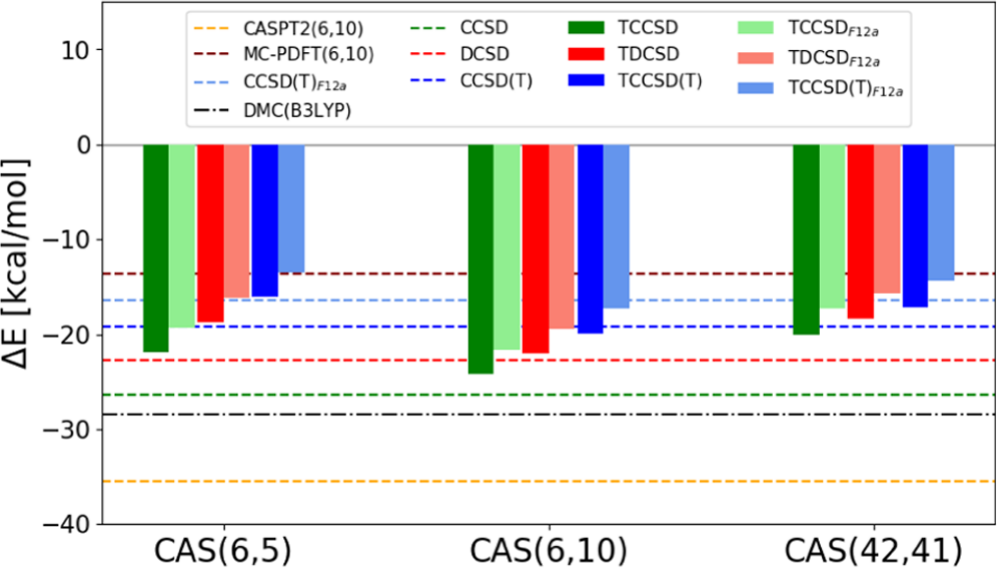
Spin gaps of [Fe(NH_3_)_6_]^2+^ over different active spaces.

The externally corrected calculations reduce the spin gap further, Figure 2, and the dependence on the active space is small compared to the active-space-only calculations. However, the tailored results from CAS(6,5) and CAS(42,41) agree better with each other, and the CAS(6,10) tailored methods are more biased toward the HS state, which is a consequence of the missing relaxation of the CAS amplitudes and can be understood as follows. In the octahedral environment, the degeneracy of the 3d orbitals is lifted resulting in the three t_2g_ and the two e_g_ orbitals. In the case of the quintet state, all 3d orbitals are occupied in the reference determinant, and in the LS state, only the t_2g_ orbitals are (doubly) occupied. Thus, the correlating d′ orbitals are especially beneficial in the LS case (as can be seen from the CASSCF (6,5) and (6,10) results) since they allow higher flexibility for the paired electrons and improve the electron correlation description. However, another important LS stabilization mechanism is related to the ligand–metal charge-transfer (CT) excitations. This mechanism has been discussed in great detail in refs (48, 104, 105). There exists a competition of excitations to the d′ orbitals, either from the occupied valence 3d orbitals or from ligand-based orbitals. By introducing the d′ orbitals into the active space and optimizing the coefficients without the ligand orbitals and then freezing the corresponding singles and doubles amplitudes at the level of the tailoring step, we artificially enhance the 3d to d′ channel, which subsequently in the tailored calculations suppresses the important ligand–metal excitations. This negatively impacts the LS state relatively to the HS state since the ligand–metal excitations, as well as the 3d → d′ excitations, are more important there compared to the HS state, and therefore the frozen singles and doubles are more harmful for the LS state.

The multiconfigurational character, measured by the largest doubles amplitude within CAS(42,41), is low: 0.041 and 0.037 for LS and HS states, respectively.

The TDCSD_*F*12*a*_ and TCCSD(T)_*F*12*a*_ results are closer to the MC-PDFT(6,10) and to the CCSD(T)_*F*12*a*_ spin gaps. The DMC and especially CASPT2 spin gap estimates are quite large compared to other methods. The CASPT2 overstabilization of the HS states has been already observed for the Fe(II)-aquo complex, and earlier by us in the context of Fe(II)-porphyrin spin gaps,^104,105^ and it has been related to the missing higher-order correlation effects that otherwise differentially stabilized the lower spin states.

#### 3.2.3 [Fe(NCH)_6_]^2+^

Now, we consider the Fe(II) compound with the NCH ligand. The largest CAS in this case is composed of 34 electrons in 31 orbitals, CAS(34,31), that contains 12 π_ML_ and 12 π_ML_^*^ orbitals (the local π bonds between the metal and the ligands), together with the five 3*d* orbitals of Fe and two σ_ML_ bonds (see the Supporting Information).

**Figure 3:**
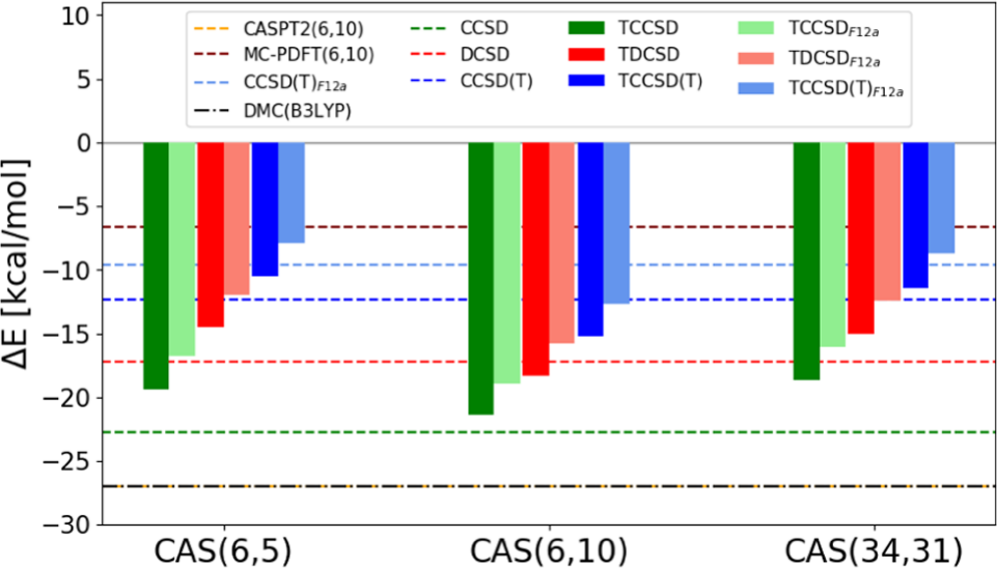
Spin gaps of [Fe(NCH)_6_]^2+^ over different active spaces.

An HS ground state is observed and electron correlation stabilizes the lower spin state (Figure 3). The results from the nontailored coupled-cluster methods substantially deviate from each other, e.g., CCSD spin gap is almost 2 times larger than the CCSD(T) one. The tailored methods agree more within each other, and in most of the cases reduce the gap further. However, the tailored results for the CAS(6,10) are again less accurate, indicating the importance of the relaxation of the amplitudes corresponding to the 3d → d′ excitations. TCCSD(T) for the largest CAS remains close to CCSD(T), while the other tailored methods are shifted noticeably more to the CCSD(T) value. As in all previous calculations, the tailored DCSD results agree well with the tailored CCSD(T) numbers, and are consistently between TCCSD and TCCSD(T) estimates. The basis set correction reduces the gap even further.

As before, the multiconfigurationality of the system is low for both spin states with the largest doubles amplitude of 0.066 and 0.038 for LS and HS states within CAS(34,31).

For this system, DMC and CASPT2 predict the most negative spin-gap (HS energetically lower than LS), while MC-PDFT predicts the least negative spin gap, with the HS state only 5 kcal/mol more stable than the LS state. Interestingly, the TCCSD(T)_*F*12*a*_ is much closer to the latter.

#### 3.2.4 [Fe(CO)_6_]^2+^

**Figure 4:**
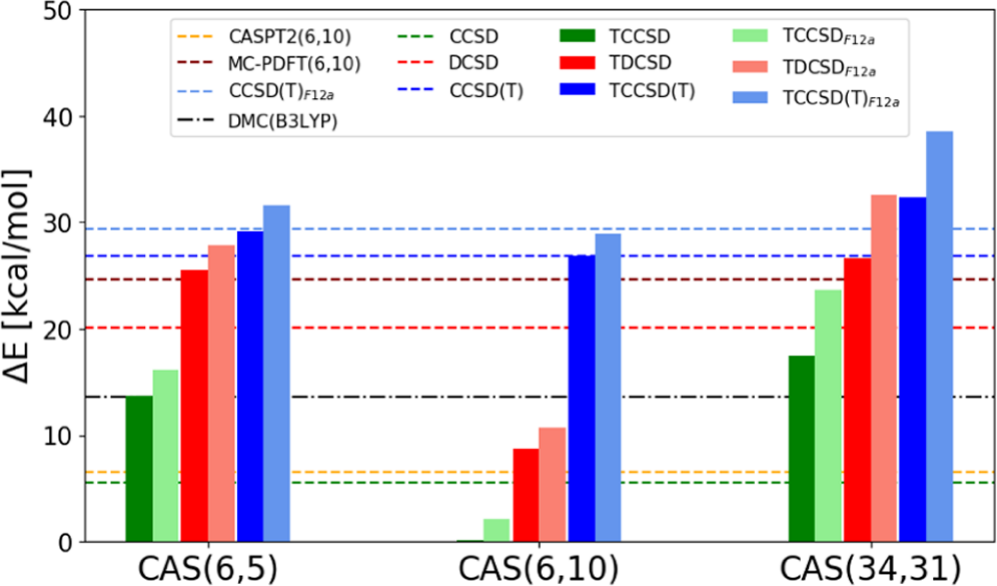
Spin gaps of [Fe(CO)_6_]^2+^ over different active spaces. The LS active space in CAS(6,10) has been obtained using state-averaged CASSCF; see main text.

In the [Fe(CO)_6_]^2+^ system, the carbon monoxide ligands strongly stabilize the low-spin state, and a singlet ground state is observed. This is to be compared to the previous cases, where an HS ground state was identified. As shown in Figure 4, the single-reference CCSD, DCSD, and CCSD(T) calculations based on ROHF orbitals give already a qualitatively correct result. CASPT2 calculated on top of CASSCF(6,10) yields a spin gap very close to the CCSD value. However, one can see large quantitative differences between methods, e.g., the gap at the CCSD(T) level is 5 times larger than at the CCSD level of theory.

The CASSCF(6,5) spin gap of −47.4 kcal/mol shows that the active space does not include all essential correlation effects that is necessary to stabilize the singlet state. The tailored approach on top of this small active space recovers the correct spin state ordering, which demonstrates that the correlation outside of this active space plays a key role in stabilizing the LS state. The tailoring noticeably improves the agreement within the CC hierarchy, with TDCSD becoming very close to TCCSD(T) (as well as to ROHF-CCSD(T)). The TCCSD gap increases by a factor of 2, compared to CCSD, and fortuitously coincides with the DMC gap. The spin gap from TDCSD for this active space is very close to the MC-PDFT (6,10) result.

The spin gaps calculated using the tailored methods on the CAS(6,10) with the double-shell d′ orbitals show a large bias towards the HS state in the TCCSD and TDCSD cases. Note that in this case, a state-averaging of HS and LS states in CASSCF was necessary to obtain an active space containing all of the d′ orbitals for the LS calculations. As shown in Figure 4, the TCCSD energies without the F12 correction are in this case nearly degenerate, and the TDCSD spin gap is much smaller than even the DCSD one. Such small spin gaps are due to the state-averaged orbitals used in the LS state and the overall less accurate frozen active space amplitudes for the LS state. This result shows how sensitive TCC could actually be to the size and quality of the chosen active space, a feature that was not observed in the previous complexes discussed in this work. The perturbative triples in TCCSD(T) account for additional relaxation effects curing some of the unbalanced description due to the active space choice. Nevertheless, large active spaces are indispensable for accurate and reliable tailored calculations to compensate for the frozen amplitudes.

For large active spaces, we use DCSD NOs to define (34,31) CAS. The active orbitals include the five 3d orbitals of the iron center, 12 π_ML_ and 12 π_ML_^*^ orbitals of the ligand, and two σ_ML_ bonds (see the Supporting Information). The CAS-CI results from this active space are still predicting the wrong order of states, as the previous CASSCF results. This can be due to the missing orbital optimization that could be added by performing a Stochastic-CASSCF optimization prior to the TCC correction. However, the tailored methods are again yielding the correct spin state order. With this large active space, the TCCSD results are closer to TDCSD and TCCSD(T), and the TDCSD value for the spin gap almost overlaps with the CCSD(T) estimate. TCCSD(T) demonstrates a further stabilization of the low-spin state.

Again, the multiconfigurational character of this complex is low as in the previous systems: the largest doubles amplitudes within CAS(34,31) are 0.053 and 0.037 for LS and HS states, respectively.

The F12 correction favors the LS state, further increasing the spin gaps. The resulting TDCSD_*F*12*a*_ and TCCSD(T)_*F*12*a*_ values for the spin gap are larger than from CCSD(T)_*F*12*a*_, in the TCCSD(T)_*F*12*a*_ case by almost 10 kcal/mol. Finally, it is worth pointing out that there is better agreement between CCSD(T), MC-PDFT, and TCCSD(T) compared to PT2 and DMC, that exhibit a bias toward the HS state. It is relevant to point out that the TCCSD(T)_*F*12*a*_ spin gap is more than 10 kcal/mol higher than the MC-PDFT result. It is possible that in this case a dependency of MC-PDFT also exists on the active space size, and that the MC-PDFT(6,10) is not converged with respect to the active space choice. Particularly relevant in this case is the correlation within the CO ligand and its direct effect on the ligand-field splitting and CT correlation.

#### 3.2.5 Overview

**Figure 5:**
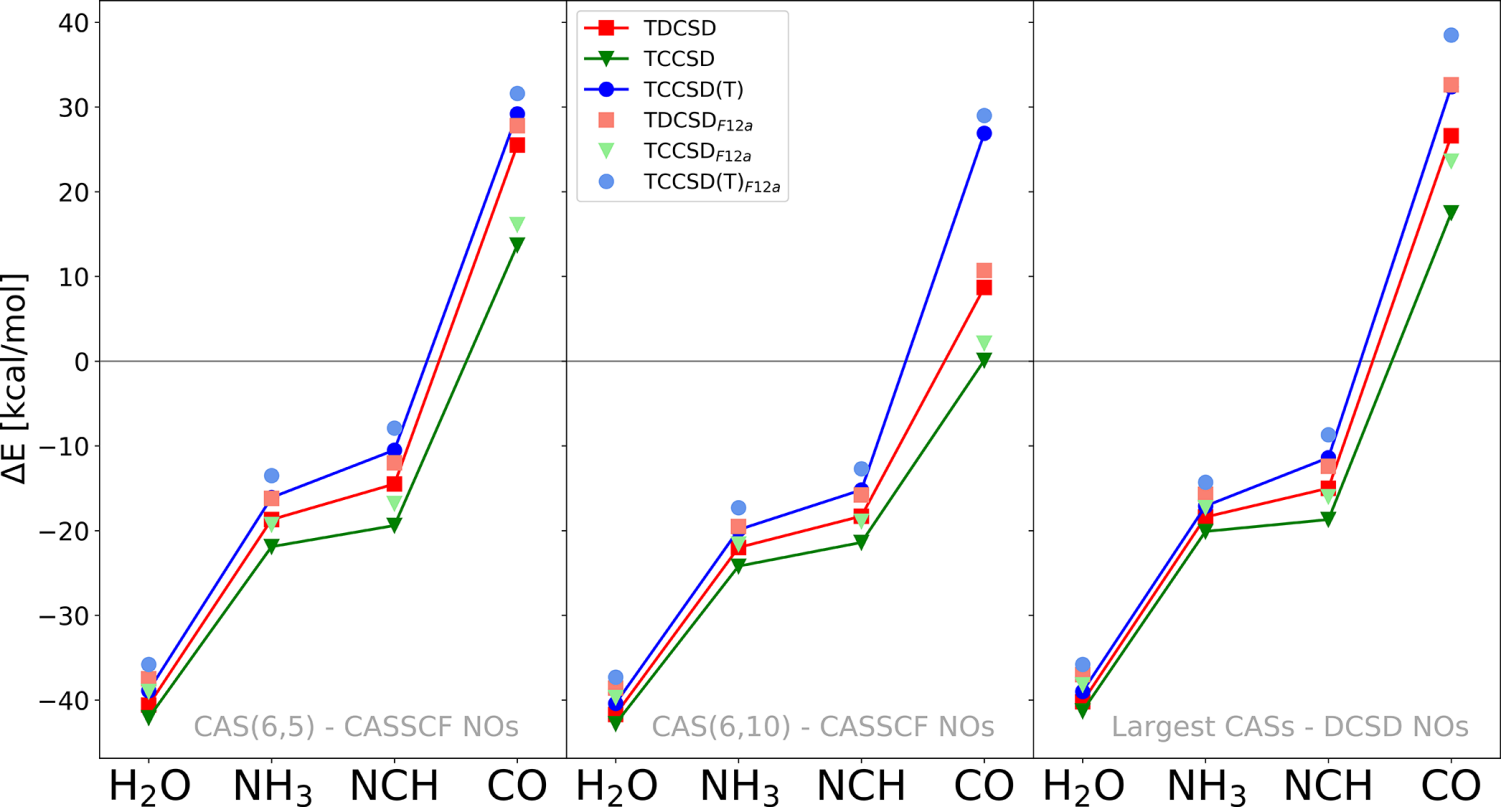
Spin gaps of the four octahedral Fe(II) complexes. Specifically, one graph for every active space.

Figure 5 offers an overview of the TCC predictions over all of the octahedral complexes presented thus far.

For all complexes, TDCSD results are between TCCSD(T) and TCCSD, and in some of the cases, they are very close to TCCSD(T) results. This suggests a higher accuracy of the TDCSD approach over TCCSD, as shown in our previous work^71^ and as expected due to previous studies on the DCSD method.^16,17^ The qualitative trend of spin gaps in these systems is well described at all coupled-cluster levels of theory and for all active spaces employed here, although the CAS (6,10) tailored results are not very accurate, especially in the case of TCCSD.

Table 1 summarizes some of the spin gap estimates available in the literature, with a focus on the variety of different methods used for the same compounds in some of the most recent studies. Very promising is the general agreement between the TCC results and MC-PDFT, showing how computationally inexpensive schemes can yield comparable accuracy or can outperform methods, such as DMC or CASPT2, commonly used for ground- and excited-state chemistry.

**Table 1:** Spin Gap Estimates of the Four Octahedral Fe(II)-Complexes in kcal/mol from Different Studies in Comparison with Some of Our Results

compound	method	Δ*E*	ref
[Fe(H_2_O)_6_]^2+^	TDCSD_*F*12*a*_	–37.0	this work
	TCCSD(T)_*F*12*a*_	–35.8	this work
	MC-PDFT	–30.7	this work
	DLPNO-CCSD(T_1_)	–33.3	(83)
	DFT-PBE[U]	–34.6	(85)
	CASPT2/CC	–42.2	(85)
	DMC	–41.0	(82)
	DMC	–58.6	(79)
[Fe(NH_3_)_6_]^2+^	TDCSD_*F*12*a*_	–15.7	this work
	TCCSD(T)_*F*12*a*_	–14.3	this work
	MC-PDFT	–13.6	this work
	DLPNO-CCSD(T_1_)	–11.3	(83)
	DFT-PBE[U]	–10.1	(85)
	CASPT2/CC	–14.9	(85)
	DMC	–28.4	(82)
	DMC	–36.7	(79)
[Fe(NCH)_6_]^2+^	TDCSD_*F*12*a*_	–12.4	this work
	TCCSD(T)_*F*12*a*_	–8.7	this work
	MC-PDFT	–6.6	this work
	DLPNO-CCSD(T_1_)	–8.8	(83)
	DFT-PBE[U]	4.8	(85)
	CASPT2/CC	–3.8	(85)
	DMC	–27.0	(82)
	DMC	–31.8	(79)
	DMC	–19.6/–21.9	(80)
[Fe(CO)_6_]^2+^	TDCSD_*F*12*a*_	32.6	this work
	TCCSD(T)_*F*12*a*_	38.5	this work
	MC-PDFT	24.6	this work
	DFT-PBE[U]	60.9	(85)
	CASPT2/CC	46.5	(85)
	DMC	13.6	(82)
	DMC	7.6	(79)

In a recent work by Mariano et al.,^85^ the spin gap estimates of these systems were calculated using a new Hubbard *U* density-corrected DFT approach. In this method, the PBE functional is evaluated on the Hubbard density, whose value *U* is obtained self-consistently. These results are shown in Table 1 together with the reference values considered in the same work, i.e., the CC-corrected CASPT2 method (CASPT2/CC), originally proposed a few years back.^106^ It has been shown by Pierloot et al.^87^ that CASPT2 yields accurate spin state energetics as long as only the valence electrons are correlated; however, an overstabilization of the higher spin states occurs in the full CASPT2 treatment, as is also evident from our CASPT2 results. The latter is found to be caused by the poor treatment of transition-metal semicore orbitals. This deficiency is mitigated in CASPT2/CC by introducing a semicore CCSD(T) correction for the (3s3p) correlation contribution. In the results shown in Table 1, the CASPT2 values used in CASPT2/CC are extrapolated to the complete basis set limit. The newly introduced DFT-PBE[U] approach has been shown in ref (85) to give the smallest mean absolute deviations for these four octahedral Fe(II) complexes in comparison to many other DFT functionals. However, it is evident from Table 1 that our spin gap estimates have a better agreement with their reference values from CASPT2/CC rather than the DFT-PBE[U] estimates (except for the L = H_2_O case). Particularly striking is the spin-gap prediction for [Fe(II)(NCH)_6_]^2+^, for which DFT-PBE[U] predicts an LS ground state, while all other methods predict an HS ground state. With the data in our hands, it is not possible to exclude that the differences in predictions are correlated to differences in the structural parameters; in fact, in this work, we employed the geometry used by Song and co-workers, allowing for direct comparison, while Mariano and co-workers used a different geometry optimization strategy.

In another recent study by Neese and co-workers,^83^ the Fe(II) complexes exhibiting a weak ligand strength, i.e., L = H_2_O, NH_3_, and NCH, have been investigated using the domain-based pair natural orbital CCSD with iterative triples (DLPNO-CCSD(T_1_)).^107^ The results shown in Table 1 represent basis set extrapolated estimates of the spin gaps. These values agree within a few kcal/mol with our TCCSD(T)_*F*12*a*_ and TDCSD_*F*12*a*_ results. Note that in the DLPNO-CCSD(T_1_) calculations scalar relativistic effects are included, which account for a further lowering of the spin gaps by 2–3 kcal/mol.^83^ Thus, the DLPNO-CCSD(T_1_) spin gaps without the relativistic correction are expected to be in between TDCSD_*F*12*a*_ and TCCSD(T)_*F*12*a*_ results.

In multiple works, DMC results have been reported (Table 1). However, all of the DMC estimates (including the values from Song et al.^82^) show a tendency to favor the HS state in comparison to our results.

This comparison with many different studies and methods demonstrates once more the difficult problem at hand. Many approaches from DFT to *ab initio* methods have been used in the past years showing a scattering of the results in a wide range of energies. In refs (83, 85), for example, many popular DFT functionals have been studied giving rise to deviations in a range up to tens of kcal/mol. Thus, even though these systems are not characterized by a strong MR character, a balanced description of their electronic structure is not trivial.

### 3.3 Fe(II)-Porphyrin

The TDC approach has also been applied to an Fe(II)-porphyrin model system. This molecular system is characterized by a number of nearly degenerate spin states. Previous studies using Stochastic-CASSCF and high-order coupled-cluster methods^104,105^ have demonstrated the stability of the intermediate state (^3^E_g_) over the high-spin state (^5^A_1g_). Furthermore, the key excitations that lead to the stabilization of the triplet state have been extensively analyzed.^48^ In a recent study, Stochastic-MRCISD calculations have shown further evidence of a correlation-induced differential stabilization of the ^3^E_g_ state over the ^5^A_1g_.^49^ In this work, we focus on the quintet–triplet spin gap Δ*E* = *E*_Q_ – *E*_T_, which is estimated to be in a range of a few kcal/mol.

The system has been investigated using four different active spaces optimized at the CASSCF level. The smallest active space (8,11) comprises five 3d orbitals of the iron atom, five empty correlating d′ orbitals (double-shell orbitals), and one σ Fe–N bonding orbital. The second smallest CAS (12,15) also contains the four Gouterman π orbitals. These π-frontier orbitals were shown to play a crucial role in stabilizing the triplet state over the quintet.^104^ The next CAS(14,18) does not correspond to a direct extension of the CAS(12,15). Instead, it contains four 4s4p Fe orbitals and the remaining three σ Fe–N bonding orbitals on top of the CAS(8,11). The four Gouterman π orbitals are not included in this case. The largest active space considered here, CAS(32,34), contains the entire π system together with the orbitals from CAS(14,18). As the size of this CAS is prohibitive for the conventional CASSCF, the Stochastic-CASSCF has been employed to optimize the orbitals. These orbitals have also been used in other works of ours and are described there in greater detail.^48,49,104,105^

The multiconfigurational character of this molecular system is more pronounced as can be seen from the largest amplitudes within the CAS(32,34): 0.242 and 0.221 for LS and HS, respectively.

**Figure 6:**
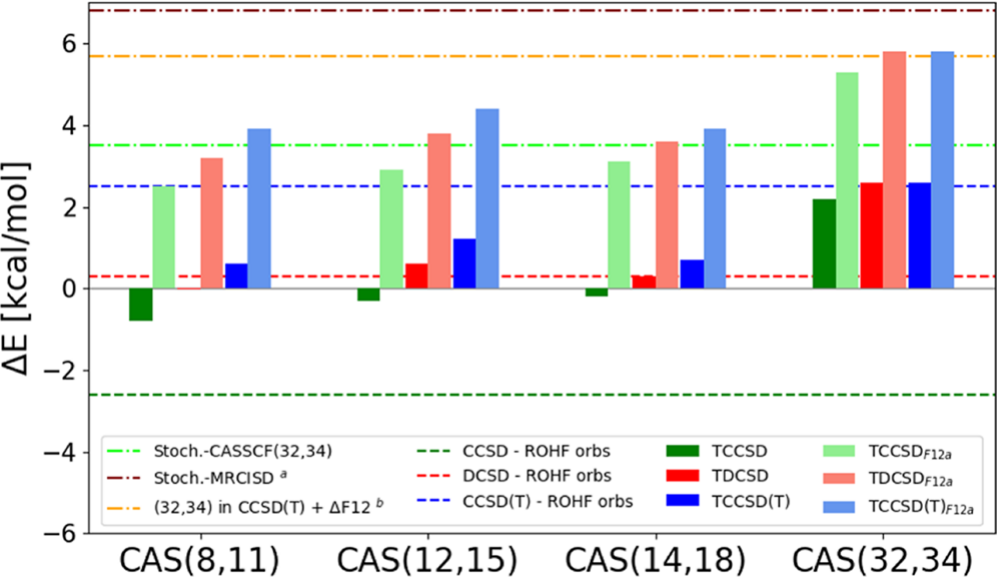
Spin gaps (Δ*E* = *E*_Q_ – *E*_T_) of the Fe(II)-porphyrin over different active spaces. ^*a*^Stochastic-MRCISD (6.8 kcal/mol) corresponds to a calculation correlating 96 electrons in 159 molecular orbitals, using CAS(32,34) as the reference wave function, ref (49). ^*b*^Our previous best estimate from a subtractive embedding scheme, ref (105).

The spin gap results are summarized in Figure 6. The tailored calculations are based on the (Stochastic-)CASSCF NOs. Additionally, the DCSD, CCSD, and CCSD(T) spin gaps calculated using ROHF orbitals are presented.

Also in this case results from the tailored methods are much closer to each other (for a chosen CAS), than from their corresponding conventional coupled-cluster methods. However, the fixed amplitudes in the small active spaces clearly reduce the accuracy of the methods, and especially the TCCSD(T) spin gaps are much smaller compared to the CCSD(T) estimate. It demonstrates that large active spaces are necessary for tailored methods to obtain reliable results. This is particularly important as the degree of multiconfigurational character increases.

In CAS(32,34) a large spin gap with triplet lower than quintet is obtained already at the Stochastic-CASSCF level. The introduction of the entire π system is essential for the triplet stabilization, while for smaller CASs, a quintet ground state is erroneously predicted. The tailored approach and the F12 correction on top of the CAS(32,34) further enlarge the spin gap prediction (5.8 kcal/mol) revealing the importance of dynamic correlation effects outside the active space and bound to the basis set incompleteness error. This result is in qualitative agreement with the recent Stochastic-MRCI on top of the same CAS(32,34), where also an enlarged spin gap has been predicted (6.8 kcal/mol).

One can compare the results of the tailored methods with a subtractive embedding employed in ref (105). The spin gap estimated using a CCSD(T)-embedded CAS(32,34)+ΔF12 amounted to 5.7 kcal/mol, and using a DCSD-embedded CAS(32,34)+ΔF12 to 3.6 kcal/mol. The tailored results are much more consistent: both TDCSD_F12*a*_ and TCCSD(T)_F12*a*_ yield 5.8 kcal/mol, in good agreement with the CCSD(T)-embedded calculation. Overall, all tailored methods for this large active space agree well with each other.

## 4 Conclusions

In this work, we have extended our explicitly correlated tailored DC/CC methods externally corrected by FCIQMC to open-shell systems and have explored their accuracy by studying Fe(II) transition-metal complexes. The active spaces have been defined using DCSD NOs, CASSCF, and Stochastic-CASSCF. Since the size of the CAS is critical to compensate for all of the potential errors arising from frozen CAS amplitudes and strong bias toward a particular reference determinant that are present in the tailored methods, we have investigated a number of active spaces, including very large ones that have been solved by the FCIQMC method. This strategy has provided the means to investigate the effects of frozen amplitudes and the SR character of the approach in spin-gap predictions.

Among the tailored schemes, the TCCSD(T) is the highest in the TCC hierarchy here investigated. The TDCSD results for the spin gaps of the four octahedral Fe(II) complexes are much closer to TCCSD(T) results than TCCSD, confirming the higher accuracy of the TDCSD method compared to TCCSD also for open-shell systems.

Comparing results across different active spaces, one can see that larger active spaces do not guarantee higher accuracy. For example, adding the correlating double-shell d′ orbitals to the active space did not improve the quality of the results, showing an evident bias toward the HS state. This is due to an artificial amplification of the 3d → d′ excitation in this frozen-amplitude approach at the expense of the important ligand to metal excitations. The overstabilization of the HS state is also observed at the level of the CASPT2 calculations. When the active space is increased in a balanced way, to include orbitals important for both states, the tailored results improve consistently for all complexes.

Remarkably, there is closer quantitative agreement between TDCSD, TCCSD(T), and MC-PDFT compared to the well-established CASPT2 and DMC correlated methods. In ref (81), the discrepancy between CASPT2 and MC-PDFT has already been reported, and same as in the present work, with the CASPT2 and MC-PDFT favoring the HS and the LS states, respectively. The semiquantitative agreement between the TCCSD(T), TDCSD, and MC-PDFT is very encouraging for these methods that are relatively cheap and can effectively be coupled to large-active-space reference wave functions.

The tailored results for the iron-porphyrin model system agree very well with our previous findings, confirming the triplet ground state in this model. The calculated TCCSD(T) and TDCSD spin gaps for our largest active space in this work (32,34) are very close to each other and to the estimate from a subtractive embedding of CAS(32,34) into CCSD(T)-F12.

The F12 correction improves the convergence toward the complete basis set limit. In all calculations presented here, it resulted in an additional LS stabilization (and triplet stabilization in Fe(II)-porphyrin case).

For all systems considered here, we found that CCSD(T) can yield quite accurate spin gap estimates, coinciding well with our best tailored results. However, even in such rather single-reference problems, a combination of coupled cluster or distinguishable cluster with FCIQMC (or alternatively, with higher-order coupled-cluster methods) provides a way for a systematic improvement of the results beyond a perturbative triples correction. We are currently working on extending the tailored methods to strongly correlated open-shell systems that usually require spin-broken reference determinants, and for which our recently developed spin-purification technique for FCIQMC^108^ will be essential.

## Supporting Information Available

Figures with all of the orbitals included in the active spaces of the four octahedral Fe(II) complexes and tables with the absolute values of all of the calculations shown in the spin-gap bar charts (PDF)
Molden data (ZIP)
