# Supplementary material for: FCIQMC-Tailored Distinguishable Cluster Approach: Open-Shell Systems
Source: J Chem Theory Comput. 2022 May 6;18(6):3427–37. doi: 10.1021/acs.jctc.2c00059 (PMC9202306; doi:10.1021/acs.jctc.2c00059)
Supplement: Supplementary file 1 — ct2c00059_si_001.pdf [file ct2c00059_si_001.pdf]

Supplemental Material: The FCIQMC Tailored Distinguishable Cluster Approach: Open-Shell Systems

Eugenio Vitale,*,† Giovanni Li Manni,*,† Ali Alavi,*,†,‡ and Daniel Kats*,†

†*Max Planck Institute for Solid State Research, Heisenbergstr. 1, 70569 Stuttgart, Germany*

‡*Department of Chemistry, University of Cambridge, Lensfield Road, Cambridge CB2 1EW, United Kingdom*

E-mail: E.Vitale@fkf.mpg.de; G.LiManni@fkf.mpg.de; A.Alavi@fkf.mpg.de; D.Kats@fkf.mpg.de

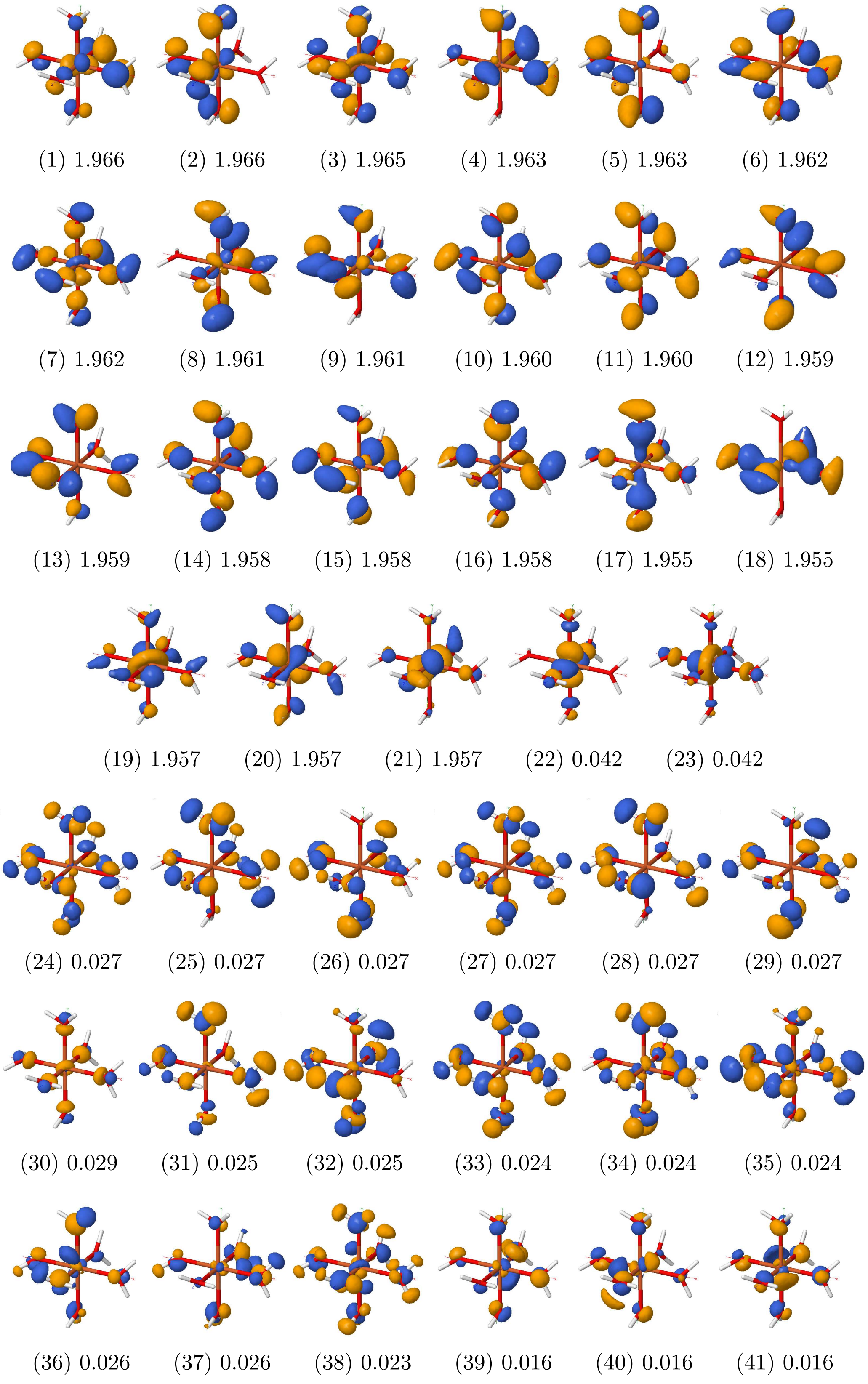

Figure S1: DCSD NOs in the CAS(42,41) for the singlet state of $[Fe(H_2O)_6]^{2+}$ and their occupation numbers.

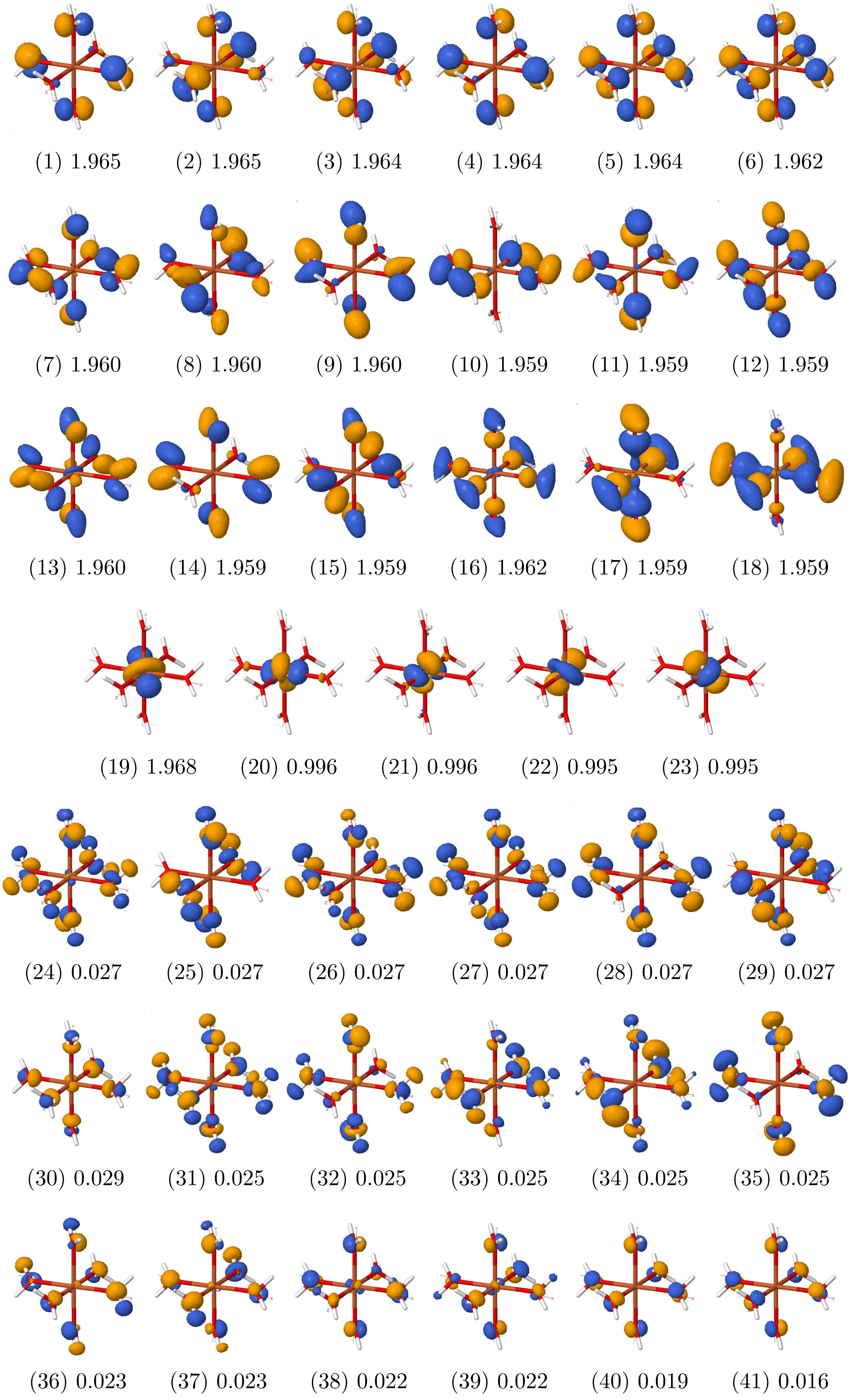

Figure S2: DCSD NOs in the CAS(42,41) for the quintet state of $[Fe(H_2O)_6]^{2+}$ and their occupation numbers.

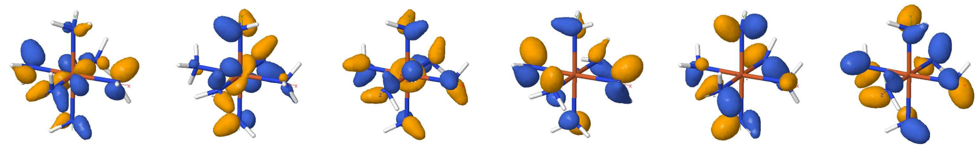

(1) 1.960 (2) 1.960 (3) 1.960 (4) 1.957 (5) 1.957 (6) 1.957

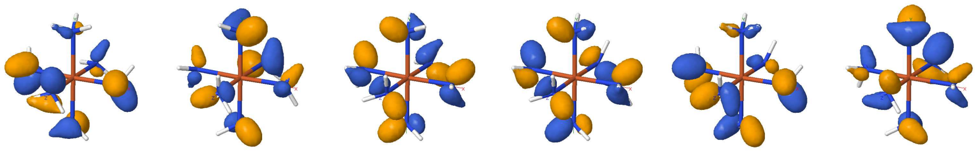

(7) 1.956 (8) 1.956 (9) 1.956 (10) 1.955 (11) 1.955 (12) 1.955

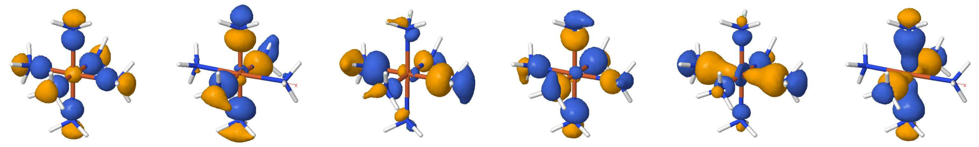

(13) 1.957 (14) 1.951 (15) 1.951 (16) 1.951 (17) 1.943 (18) 1.943

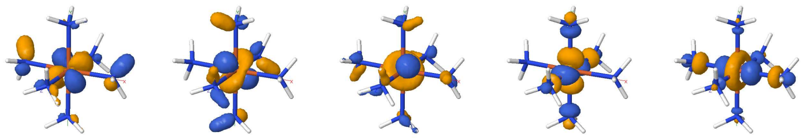

(19) 1.953 (20) 1.953 (21) 1.953 (22) 0.048 (23) 0.048

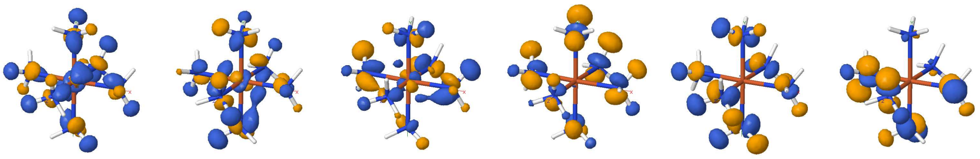

(24) 0.030 (25) 0.030 (26) 0.030 (27) 0.027 (28) 0.027 (29) 0.027

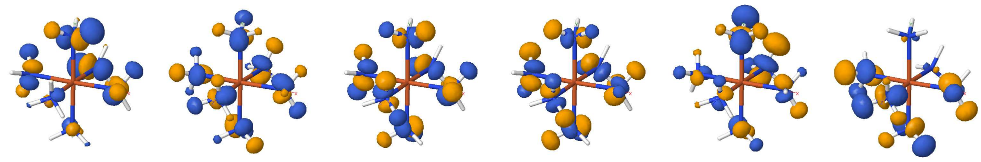

(30) 0.027 (31) 0.027 (32) 0.026 (33) 0.026 (34) 0.026 (35) 0.026

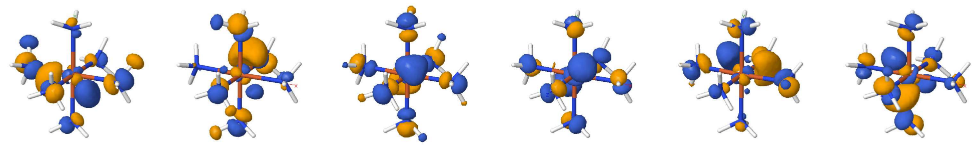

(36) 0.023 (37) 0.023 (38) 0.023 (39) 0.022 (40) 0.022 (41) 0.022

Figure S3: DCSD NOs in the CAS(42,41) for the singlet state of $[Fe(NH_3)_6]^{2+}$ and their occupation numbers.

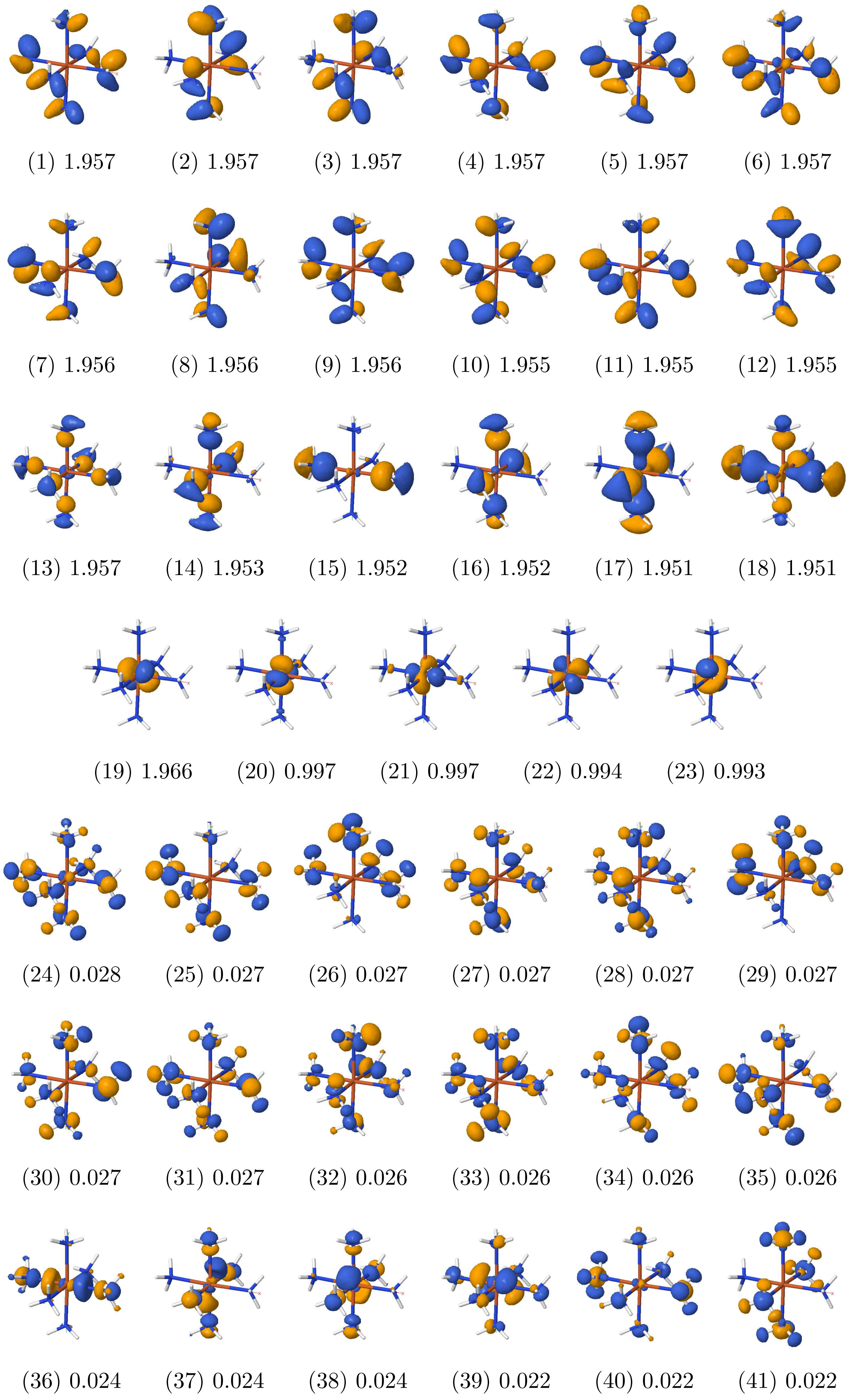

Figure S4: DCSD NOs in the CAS(42,41) for the quintet state of $[Fe(NH_3)_6]^{2+}$ and their occupation numbers.

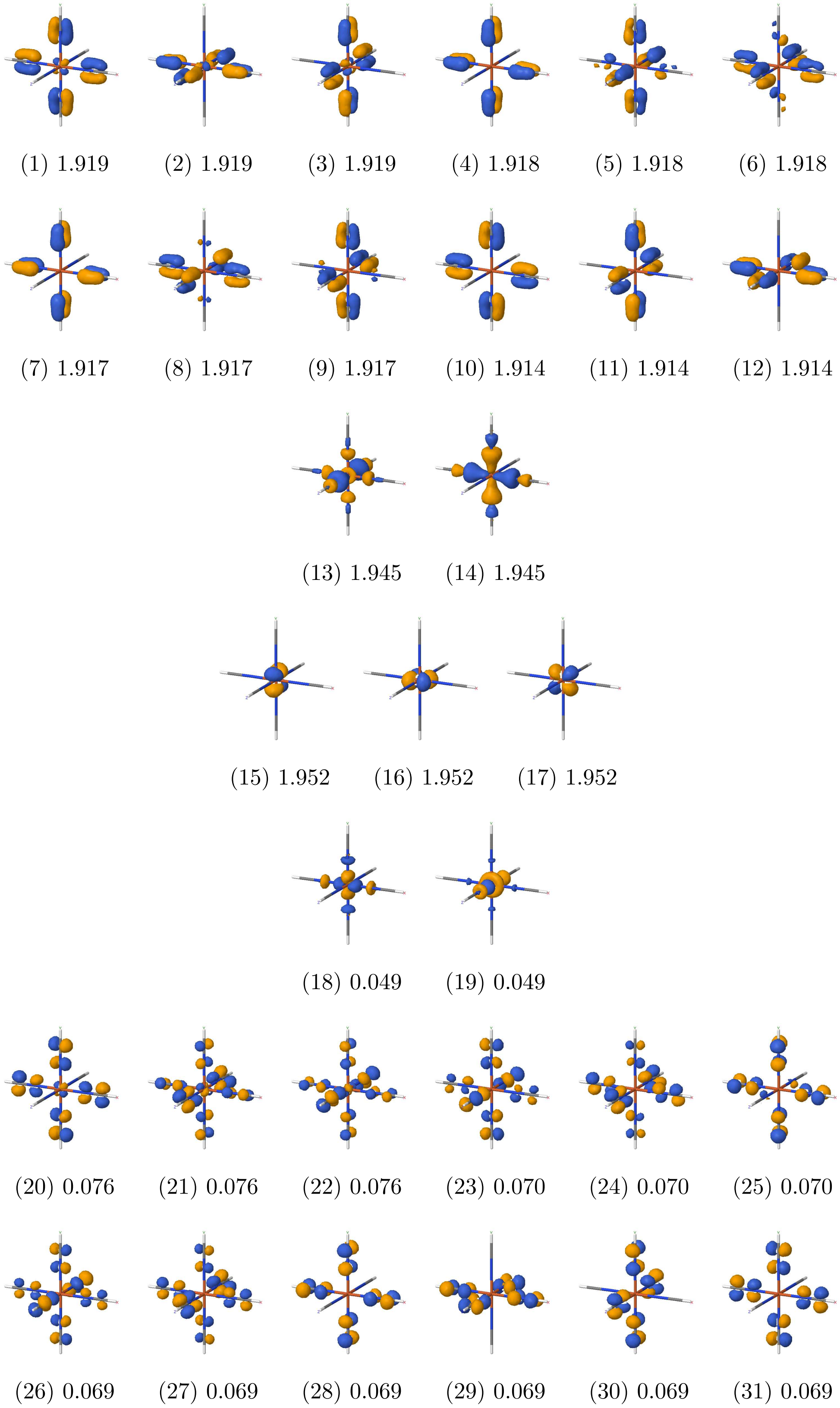

Figure S5: DCSD NOs in the CAS(34,31) for the singlet state of $[Fe(NCH)_6]^{2+}$ and their occupation numbers.

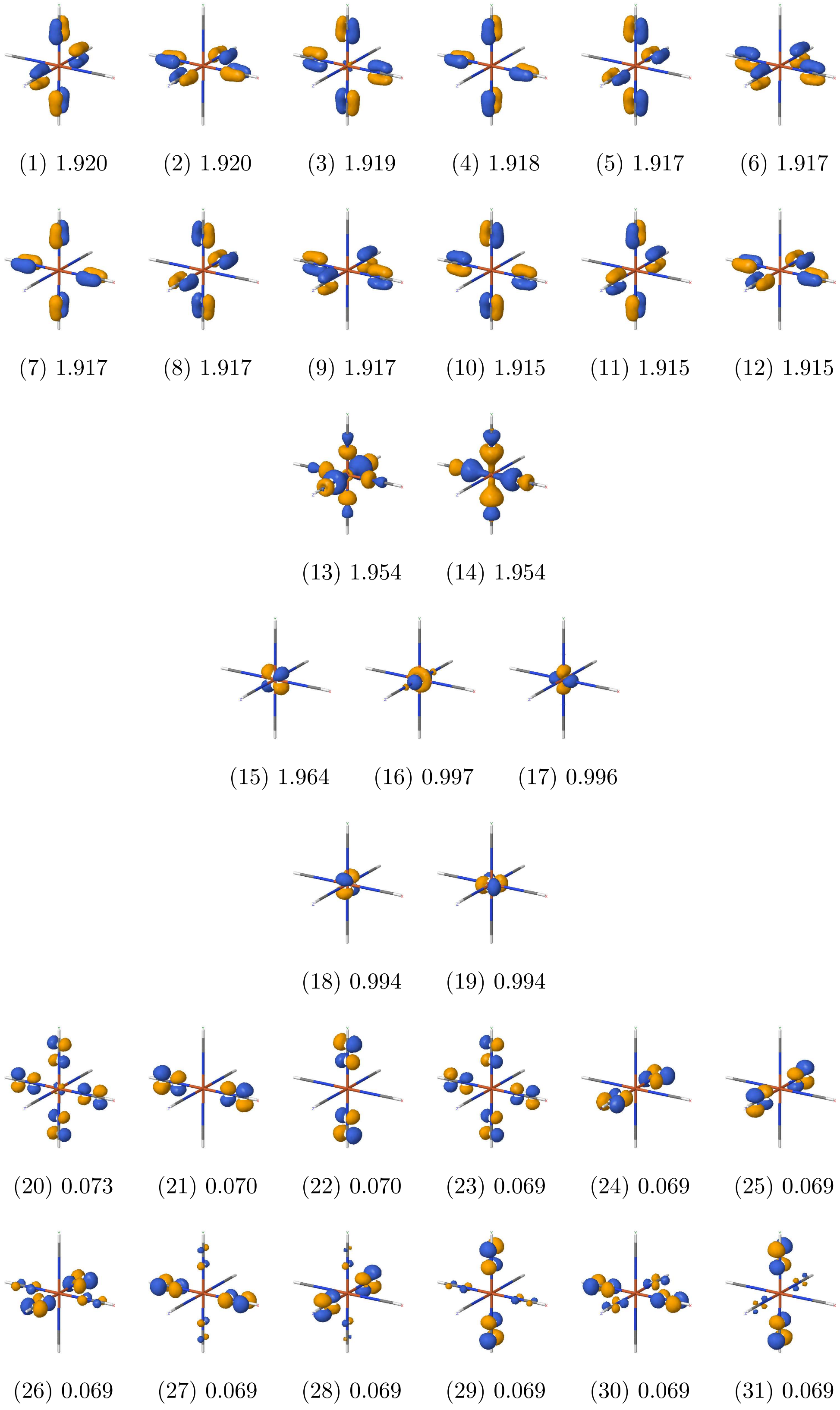

Figure S6: DCSD NOs in the CAS(34,31) for the quintet state of $[Fe(NCH)_6]^{2+}$ and their occupation numbers.

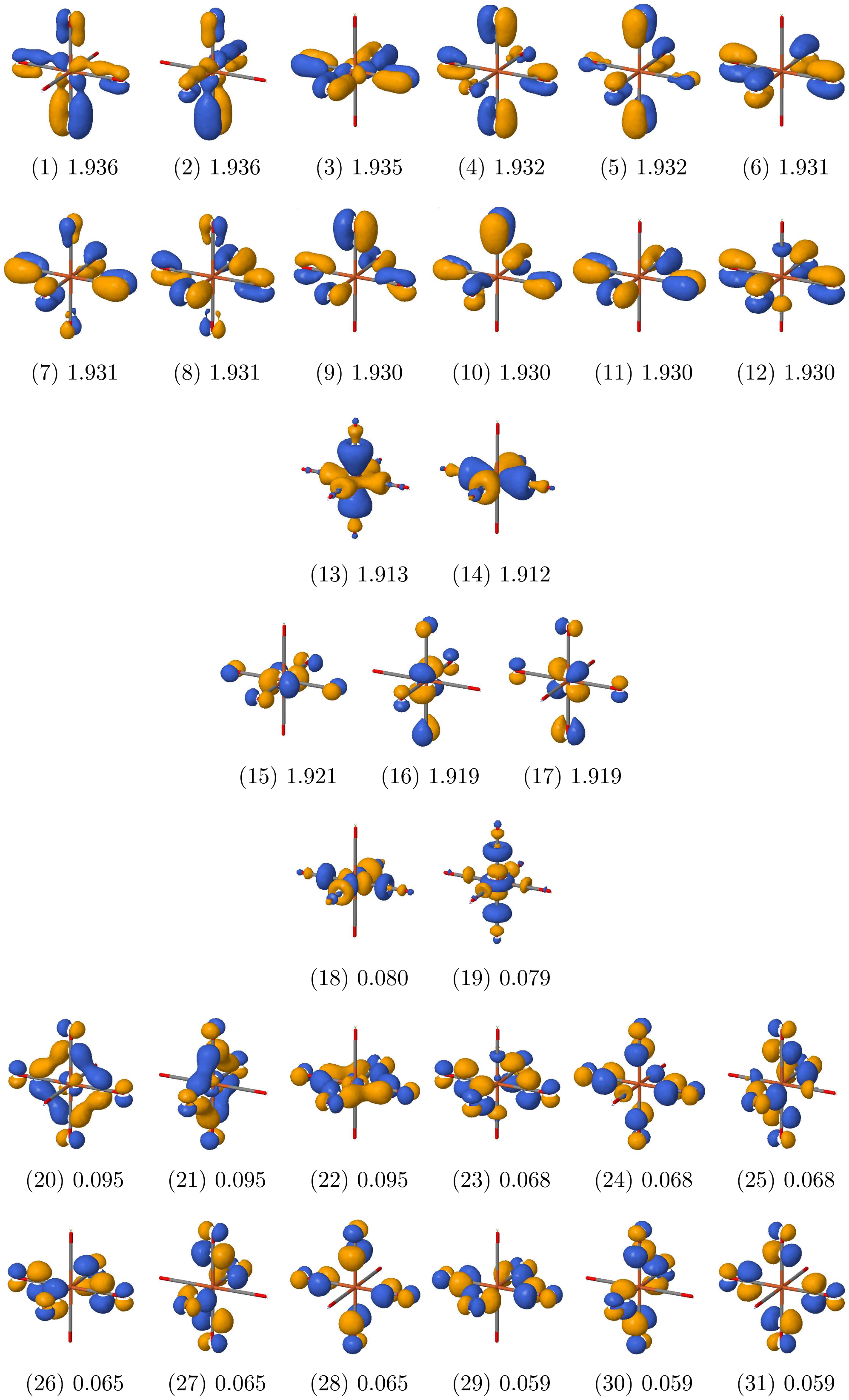

Figure S7: DCSD NOs in the CAS(34,31) for the singlet state of $[Fe(CO)_6]^{2+}$ and their occupation numbers.

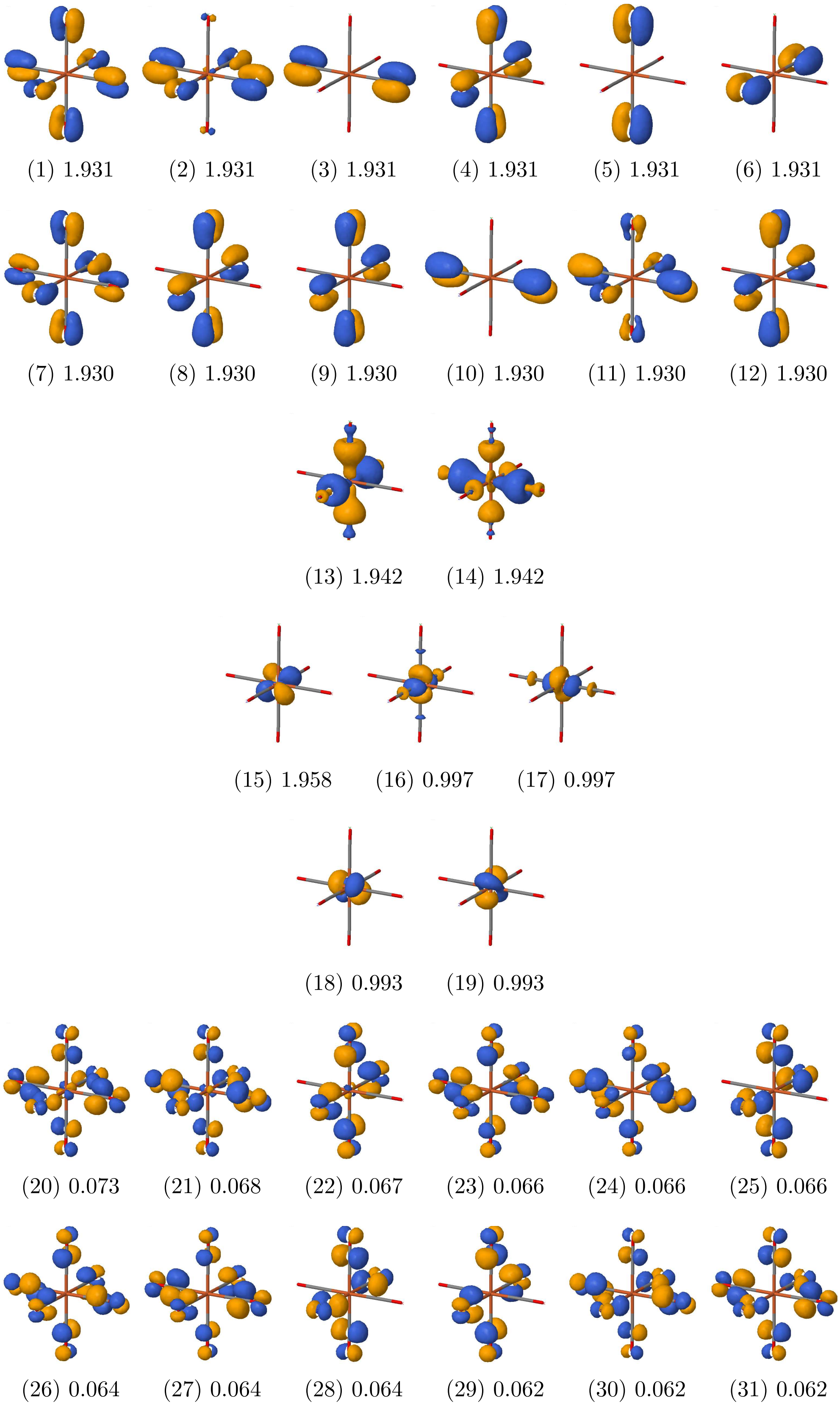

Figure S8: DCSD NOs in the CAS(34,31) for the quintet state of $[Fe(CO)_6]^{2+}$ and their occupation numbers.

Table 1: Energies of the low-spin (LS) and high-spin (HS) states of $[Fe(H_2O)_6]^{2+}$ in Hartree and the spin gap ΔE = HS-LS in kcal/mol for different active spaces and NOs.

Method	LS	HS	ΔE
	**ROHF orbitals**		
DCSD	-1720.5554	-1720.6253	**-43.9**
CCSD	-1720.5095	-1720.5831	**-46.2**
CCSD(T)	-1720.5783	-1720.6441	**-41.3**
	**CAS(6,5) - CASSCF NOs**		
CASSCF(6,5)	-1718.3734	-1718.4801	**-67.0**
CASPT2	-1720.4210	-1720.5070	**-54.0**
MC-PDFT	-1721.1773	-1721.2232	**-28.8**
DCSD	-1720.5554	-1720.6253	**-43.9**
TDCSD	-1720.5607	-1720.6253	**-40.6**
TCCSD	-1720.5160	-1720.5831	**-42.1**
TCCSD(T)	-1720.5820	-1720.6441	**-38.9**
$DCSD_{F12a}$	-1720.8336	-1720.8984	**-40.7**
$TDCSD_{F12a}$	-1720.8387	-1720.8984	**-37.5**
$TCCSD_{F12a}$	-1720.7946	-1720.8568	**-39.0**
$TCCSD(T)_{F12a}$	-1720.8606	-1720.9177	**-35.8**
	**CAS(6,10) - CASSCF NOs**		
CASSCF(6,10)	-1718.4558	-1718.5448	**-55.8**
CASPT2	-1720.4025	-1720.4893	**-54.5**
MC-PDFT	-1721.1439	-1721.1928	**-30.7**
DCSD	-1720.5550	-1720.6251	**-43.9**
TDCSD	-1720.5622	-1720.6286	**-41.7**
TCCSD	-1720.5196	-1720.5879	**-42.8**
TCCSD(T)	-1720.5819	-1720.6463	**-40.4**
$DCSD_{F12a}$	-1720.8331	-1720.8981	**-40.7**
$TDCSD_{F12a}$	-1720.8401	-1720.9016	**-38.6**
$TCCSD_{F12a}$	-1720.7981	-1720.8614	**-39.7**
$TCCSD(T)_{F12a}$	-1720.8604	-1720.9198	**-37.3**
	**CAS(42,41) - DCSD NOs**		
FCIQMC(42,41)	-1718.7627	-1718.8639	**-63.5**
DCSD	-1720.5513	-1720.6224	**-44.6**
TDCSD	-1720.5853	-1720.6493	**-40.2**
TCCSD	-1720.5522	-1720.6180	**-41.3**
TCCSD(T)	-1720.6004	-1720.6625	**-39.0**
$DCSD_{F12a}$	-1720.8287	-1720.8948	**-41.5**
$TDCSD_{F12a}$	-1720.8625	-1720.9215	**-37.0**
$TCCSD_{F12a}$	-1720.8299	-1720.8907	**-38.2**
$TCCSD(T)_{F12a}$	-1720.8781	-1720.9352	**-35.8**
DMC(B3LYP)[?]	-1722.1692	-1722.2347	**-41.0**

Table 2: **Energies of the low-spin (LS) and high-spin (HS) states of $[Fe(NH_3)_6]^{2+}$ in Hartree and the spin gap ΔE = HS-LS in kcal/mol for different active spaces and NOs.**

Method	LS	HS	ΔE
	**ROHF orbitals**		
DCSD	-1601.4893	-1601.5255	**-22.7**
CCSD	-1601.4420	-1601.4841	**-26.4**
CCSD(T)	-1601.5168	-1601.5473	**-19.2**
	**CAS(6,5) - CASSCF NOs**		
CASSCF(6,5)	-1599.3871	-1599.4841	**-60.9**
CASPT2	-1601.3154	-1601.3705	**-34.6**
MC-PDFT	-1602.0610	-1602.0814	**-12.8**
DCSD	-1601.4894	-1601.5255	**-22.6**
TDCSD	-1601.4941	-1601.5240	**-18.7**
TCCSD	-1601.4479	-1601.4828	**-21.9**
TCCSD(T)	-1601.5201	-1601.5458	**-16.1**
$DCSD_{F12a}$	-1601.7179	-1601.7497	**-19.9**
$TDCSD_{F12a}$	-1601.7225	-1601.7482	**-16.2**
$TCCSD_{F12a}$	-1601.6768	-1601.7076	**-19.3**
$TCCSD(T)_{F12a}$	-1601.7490	-1601.7706	**-13.5**
	**CAS(6,10) - CASSCF NOs**		
CASSCF(6,10)	-1599.4734	-1599.5500	**-48.1**
CASPT2	-1601.2953	-1601.3519	**-35.5**
MC-PDFT	-1602.0296	-1602.0512	**-13.6**
DCSD	-1601.4888	-1601.5251	**-22.8**
TDCSD	-1601.4933	-1601.5284	**-22.0**
TCCSD	-1601.4501	-1601.4887	**-24.2**
TCCSD(T)	-1601.5174	-1601.5491	**-19.9**
$DCSD_{F12a}$	-1601.7172	-1601.7492	**-20.1**
$TDCSD_{F12a}$	-1601.7215	-1601.7525	**-19.5**
$TCCSD_{F12a}$	-1601.6789	-1601.7133	**-21.6**
$TCCSD(T)_{F12a}$	-1601.7462	-1601.7738	**-17.3**
	**CAS(42,41) - DCSD NOs**		
FCIQMC(42,41)	-1599.7477	-1599.8114	**-40.0**
DCSD	-1601.4838	-1601.5228	**-24.5**
TDCSD	-1601.5194	-1601.5487	**-18.4**
TCCSD	-1601.4845	-1601.5165	**-20.1**
TCCSD(T)	-1601.5364	-1601.5636	**-17.1**
DCSD-F12a	-1601.7123	-1601.7469	**-21.7**
TDCSD-F12a	-1601.7476	-1601.7726	**-15.7**
TCCSD-F12a	-1601.7132	-1601.7408	**-17.3**
$TCCSD(T)_{F12a}$	-1601.7650	-1601.7879	**-14.3**
DMC(B3LYP)[?]	-1602.9807	-1603.0257	**-28.4**

Table 3: Energies of the low-spin (LS) and high-spin (HS) states of $[Fe(NCH)_6]^{2+}$ in Hartree and the spin gap ΔE = HS-LS in kcal/mol for different active spaces and NOs.

Method	LS	HS	ΔE
	**ROHF orbitals**		
DCSD	-1822.2619	-1822.2893	**-17.2**
CCSD	-1822.1693	-1822.2054	**-22.7**
CCSD(T)	-1822.3070	-1822.3266	**-12.3**
	**CAS(6,5) - CASSCF NOs**		
CASSCF(6,5)	-1819.5024	-1819.6138	**-69.9**
CASPT2	-1822.1028	-1822.1441	**-25.9**
MC-PDFT	-1823.0020	-1823.0148	**-8.0**
DCSD	-1822.2621	-1822.2893	**-17.1**
TDCSD	-1822.2662	-1822.2893	**-14.5**
TCCSD	-1822.1745	-1822.2054	**-19.4**
TCCSD(T)	-1822.3098	-1822.3266	**-10.5**
$DCSD_{F12a}$	-1822.5566	-1822.5796	**-14.5**
$TDCSD_{F12a}$	-1822.5606	-1822.5796	**-12.0**
$TCCSD_{F12a}$	-1822.4700	-1822.4968	**-16.8**
$TCCSD(T)_{F12a}$	-1822.6053	-1822.6179	**-7.9**
	**CAS(6,10) - CASSCF NOs**		
CASSCF(6,10)	-1819.5930	-1819.6798	**-54.4**
CASPT2	-1822.0830	-1822.1262	**-27.1**
MC-PDFT	-1822.9747	-1822.9852	**-6.6**
DCSD	-1822.2610	-1822.2889	**-17.5**
TDCSD	-1822.2627	-1822.2918	**-18.3**
TCCSD	-1822.1755	-1822.2096	**-21.4**
TCCSD(T)	-1822.3037	-1822.3279	**-15.2**
$DCSD_{F12a}$	-1822.5553	-1822.5792	**-15.0**
$TDCSD_{F12a}$	-1822.5568	-1822.5820	**-15.8**
$TCCSD_{F12a}$	-1822.4707	-1822.5009	**-18.9**
$TCCSD(T)_{F12a}$	-1822.5990	-1822.6191	**-12.7**
	**CAS(34,31) - DCSD NOs**		
FCIQMC(34,31)	-1819.9611	-1820.0747	**-71.3**
DCSD	-1822.2514	-1822.2831	**-19.9**
TDCSD	-1822.3029	-1822.3269	**-15.0**
TCCSD	-1822.2405	-1822.2703	**-18.7**
TCCSD(T)	-1822.3276	-1822.3457	**-11.4**
DCSD-F12a	-1822.5455	-1822.5730	**-17.3**
TDCSD-F12a	-1822.5967	-1822.6165	**-12.4**
TCCSD-F12a	-1822.5351	-1822.5607	**-16.0**
$TCCSD(T)_{F12a}$	-1822.6222	-1822.6361	**-8.7**
DMC(B3LYP)[?]	-1824.1583	-1824.2014	**-27.0**

Table 4: Energies of the low-spin (LS) and high-spin (HS) states of $[Fe(CO)_6]^{2+}$ in Hartree and the spin gap ΔE = HS-LS in kcal/mol for different active spaces and NOs.

Method	LS	HS	ΔE
	**ROHF orbitals**		
DCSD	-1941.3995	-1941.3674	**20.1**
CCSD	-1941.2925	-1941.2836	**5.6**
CCSD(T)	-1941.4496	-1941.4068	**26.9**
	**CAS(6,5) - CASSCF NOs**		
CASSCF(6,5)	-1938.5142	-1938.5898	**-47.4**
CASPT2	-1941.2573	-1941.2473	**6.3**
MC-PDFT	-1942.1571	-1942.1324	**15.5**
DCSD	-1941.3987	-1941.3674	**19.6**
TDCSD	-1941.4081	-1941.3674	**25.5**
TCCSD	-1941.3054	-1941.2836	**13.7**
TCCSD(T)	-1941.4533	-1941.4068	**29.2**
$DCSD_{F12a}$	-1941.7306	-1941.6955	**22.0**
$TDCSD_{F12a}$	-1941.7398	-1941.6955	**27.8**
$TCCSD_{F12a}$	-1941.6383	-1941.6127	**16.1**
$TCCSD(T)_{F12a}$	-1941.7862	-1941.7358	**31.6**
	**CAS(6,10) - CASSCF NOs**		
CASSCF(6,10)	-1938.5317	-1938.6576	**-79.0**
CASPT2	-1941.2385	-1941.2281	**6.5**
MC-PDFT	-1942.1423	-1942.1031	**24.6**
DCSD	-1941.3981	-1941.3670	**19.5**
TDCSD	-1941.3835	-1941.3697	**8.7**
TCCSD	-1941.2879	-1941.2878	**0.1**
TCCSD(T)	-1941.4506	-1941.4077	**26.9**
$DCSD_{F12a}$	-1941.7293	-1941.7026	**16.7**
$TDCSD_{F12a}$	-1941.7146	-1941.6976	**10.7**
$TCCSD_{F12a}$	-1941.6200	-1941.6167	**2.1**
$TCCSD(T)_{F12a}$	-1941.7827	-1941.7366	**29.0**
	**CAS(34,31) - DCSD NOs**		
FCIQMC(34,31)	-1938.9488	-1939.0615	**-70.7**
DCSD	-1941.3832	-1941.3584	**15.6**
TDCSD	-1941.4568	-1941.4143	**26.6**
TCCSD	-1941.3826	-1941.3547	**17.5**
TCCSD(T)	-1941.4835	-1941.4318	**32.4**
$DCSD_{F12a}$	-1941.7198	-1941.6856	**21.5**
$TDCSD_{F12a}$	-1941.7930	-1941.7411	**32.6**
$TCCSD_{F12a}$	-1941.7198	-1941.6823	**23.6**
$TCCSD(T)_{F12a}$	-1941.8207	-1941.7594	**38.5**
DMC(B3LYP)[?]	-1943.3449	-1943.3232	**13.6**

Table 5: Energies of the ^3E_g and $^5A_{1g}$ states of Fe(II)-porphyrin in Hartree and the spin gap ΔE in kcal/mol for different active spaces using CASSCF NOs.

Method	^3E_g	$^5A_{1g}$	ΔE
**ROHF orbitals**			
DCSD	-1954.4357	-1954.4352	**0.3**
CCSD	-1954.3226	-1954.3267	**-2.6**
CCSD(T)	-1954.5247	-1954.5207	**2.5**

**CAS(8,11)**				**CAS(12,15)**			
CASSCF(8,11)	-1951.0259	-1951.0412	**-9.6**	CASSCF(12,15)	-1951.0678	-1951.0830	**-9.6**
MC-PDFT	-1956.3172	-1956.2903	**16.9**	MC-PDFT	-1956.3112	-1956.2817	**18.5**
DCSD	-1954.4336	-1954.4343	**-0.4**	DCSD	-1954.4334	-1954.4341	**-0.5**
CCSD	-1954.3201	-1954.3256	**-3.5**	CCSD	-1954.3198	-1954.3255	**-3.5**
TDCSD	-1954.4358	-1954.4358	**-0.0**	TDCSD	-1954.4465	-1954.4456	**0.6**
TCCSD	-1954.3292	-1954.3304	**-0.8**	TCCSD	-1954.3442	-1954.3448	**-0.3**
TCCSD(T)	-1954.5205	-1954.5195	**0.6**	TCCSD(T)	-1954.5245	-1954.5226	**1.2**
$DCSD_{F12a}$	-1954.8679	-1954.8634	**2.8**	$DCSD_{F12a}$	-1954.8677	-1954.8632	**2.8**
$TDCSD_{F12a}$	-1954.8700	-1954.8650	**3.2**	$TDCSD_{F12a}$	-1954.8807	-1954.8746	**3.8**
$TCCSD_{F12a}$	-1954.7646	-1954.7607	**2.5**	$TCCSD_{F12a}$	-1954.7795	-1954.7750	**2.9**
$TCCSD(T)_{F12a}$	-1954.9559	-1954.9498	**3.9**	$TCCSD(T)_{F12a}$	-1954.9598	-1954.9528	**4.4**
**CAS(14,18)**				**CAS(32,34)**			
CASSCF(14,18)	-1951.0848	-1951.0859	**-0.7**	Stoch.-CASSCF(32,34)	-1951.3590	-1951.3530	**-3.8**
MC-PDFT	-1956.3152	-1956.2914	**15.0**	MC-PDFT	-1956.2650	-1956.2417	**14.6**
DCSD	-1954.4331	-1954.4341	**-0.6**	DCSD	-1954.4310	-1954.4326	**-1.0**
CCSD	-1954.3196	-1954.3255	**-3.7**	CCSD	-1954.3169	-1954.3235	**-4.2**
TDCSD	-1954.4436	-1954.4431	**0.3**	TDCSD	-1954.4992	-1954.4950	**2.6**
TCCSD	-1954.3382	-1954.3384	**-0.2**	TCCSD	-1954.4093	-1954.4059	**2.2**
TCCSD(T)	-1954.5255	-1954.5244	**0.7**	TCCSD(T)	-1954.5499	-1954.5458	**2.6**
$DCSD_{F12a}$	-1954.8673	-1954.8630	**2.7**	$DCSD_{F12a}$	-1954.8648	-1954.8613	**2.2**
$TDCSD_{F12a}$	-1954.8776	-1954.8719	**3.6**	$TDCSD_{F12a}$	-1954.9324	-1954.9231	**5.8**
$TCCSD_{F12a}$	-1954.7733	-1954.7684	**3.1**	$TCCSD_{F12a}$	-1954.8435	-1954.8350	**5.3**
$TCCSD(T)_{F12a}$	-1954.9606	-1954.9543	**3.9**	$TCCSD(T)_{F12a}$	-1954.9841	-1954.9750	**5.8**
